# Supplementary material for: The effect of carbon fertilization on naturally regenerated and planted US forests
Source: Nat Commun. 2022 Sep 19;13:5490. doi: 10.1038/s41467-022-33196-x (PMC9485135; doi:10.1038/s41467-022-33196-x)
Supplement: Supplementary file 1 — Supplementary Information [file 41467_2022_33196_MOESM1_ESM.pdf]

Supplementary information

Title

The Effect of Carbon Fertilization on Naturally Regenerated and Planted U.S. Forests

Authors

Eric C. Davis, Brent Sohngen, and David J. Lewis

This file includes:

Supplementary Tables 1 to 10

Supplementary Table 1. Definitions of key PRISM variables

	PRISM Variables *PRISM Climate Group, 2019*
Seasonal Temperature and Precipitation	Variables were transformed into lifetime seasonal averages. The method employed was somewhat involved. The PRISM data only goes back to 1895. As in our study we have stands up to 100 years of age and as we want to understand the effect of the climate conditions under which they grew by comparing stands observed in 1960 to those observed in 2017, we ideally would have had climate data going back as far as 1861. To overcome this issue of 34 years of missing data, we took an average of the climate conditions that existed in each county between 1895 and 1914. As anthropogenic climate impacts were minimal in those years, using this twenty year average for each of the missing years felt sufficient to well approximate the conditions that existed in those years while also having the advantage that it prevented the problem of potentially basing missing year values on an outlier year. That might have occurred had we chosen only one year upon which to base our calculations for the missing years. With the needed climate data established, we then calculated the lifetime average seasonal temperature and precipitation for each stand age. The seasons were defined as follows: Winter (December through February), Spring (March through May), Summer (June through August), and Autumn (September through November).
Seasonal Vapor Pressure Deficit	Variables for both the average seasonal and average yearly minimum/maximum vapor pressure deficit were created. The seasons were defined as above.
Mean Growing Season Months	Variable indicates the lifetime annual mean for the number of months where the average monthly temperature was greater than 0C.
Number of months with mean temperature >30/35C	Variable was created that records the lifetime number of months where the average maximum monthly temperature was greater than a specified threshold, either 30C or 35C.

Supplementary Table 2. Definitions of key USFS-FIA variables

	USFS-FIA Variables	*Burrell et al., 2018*
Natural Log of Volume per Hectare	Variable is a logarithmic transformation of the average net volume of wood in cubic meters per hectare in the central stem of trees. These data are derived from breast-height volume measurements of sample trees that are transformed using techniques that rely on the USFS' sampling design to enable estimation of the theoretical number of trees and therefore volume per hectare on each plot.	
Natural Log of Lifetime CO2 Exposure	Variable is a logarithmic transformation of the sum of yearly atmospheric CO_2 exposure over the lifetime of the stand. The CO_2 values are those for the years the stand lived based on the age recorded by the USFS.	
1/Stand Age	Variable is the inverse of the field-recorded stand age.	
Regrowth Method	Dummy variable that is equal to 1 if the stand was artificially regenerated. "An artificially regenerated stand is established by planting or artificial seeding."	
Site Class	Variable based on "a classification of forest land in terms of inherent capacity to<br>grow crops of industrial wood. Identifies the potential growth in cubic feet/acre/year and is based on the culmination of mean annual increment of fully stocked natural stands."<br>Class 1: >15.7 cubic meters/hectare/year<br>Class 2: 11.5-15.7 cubic meters/hectare/year<br>Class 3: 8.4-11.4 cubic meters/hectare/year<br>Class 4: 5.9-8.3 cubic meters/hectare/year<br>Class 5: 3.5-5.8 cubic meters/hectare/year<br>Class 6: 1.4-3.4 cubic meters/hectare/year<br>One issue that was encountered was the lack of data for land of the most productive types. This limitation means that our findings are most robust for Class 3 to 6 forest sites.	
Elevation	Variable for the distance above sea level a plot is located.	
Aspect	Variable included in analysis due to relevance identified by Måren et al. (2015) and identifies "the direction of the slope, to the nearest degree. North is recorded as 360. When slope is <5º, there is no aspect and this item is set to zero." Plots with an aspect between 87.5º and 92.5º and between 267.5º and 272.5º were dropped from the study to ensure that the north and south groups had a more than nominal difference. Then, the variable was formulated as a series of dummy variables for north-facing, south-facing and non-sloped plots.	
Physiographic Class	Variable depicts "the general effect of land form, topographical position, and soil on moisture available to trees." There are three major types of sites: 1) Xeric sites that are normally low or deficient in available moisture, 2) Mesic sites that normally have adequate available moisture, and 3) Hydric sites that normally have abundant or overabundant moisture all year. As xeric hydrologic conditions have been found to limit growth (Newingham et al., 2013), physiographic conditions were controlled for through the use of three dummy variables for xeric, mesic and hydric soils.	
Slope	The angle of slope, in percent, of the condition. Valid values are 000 through 155 and were measured by the USFS by sighting along the average incline or decline of the condition.	
Ownership	Variable for the class in which the landowner belongs.<br>Public category includes:<br>11) National Forest 12) National Grassland and/or Prairie<br>13) Other Forest Service Land 21) National Park Service<br>22) Bureau of Land Management 23) Fish and Wildlife Service<br>24) Departments of Defense/Energy 25) Other Federal<br>31) State including State universities 32) Local including water authorities<br>33) Other non-federal public<br>Private category references:<br>46) Undifferentiated private and Native American	
Stocking Density	Variable indicating how densely stocked are the trees on the plot. 1) Overstocked (100+%) /<br>2) Fully Stocked (60-99%) / 3) Medium Stocked (35-59%) / 4) Poorly Stocked (10-34%) /<br>5) Non-stocked (0-9%)	
Disturbances	Variable "indicating the kind of disturbance that has occurred since the last measurement or within the last 5 years for new plots. The area affected by the disturbance must be at least 1 acre in size. A significant level of disturbance (mortality or damage to 25% of the trees in the condition) is required."	
Time Dummies	Variable constituted in two ways. First, as two groups: 1) the observations that were made up to 1990 & 2) those made after 2000. Secondly, the evaluations made from 2000 onward, the ones made between 1981 & 1990, & the ones made up to 1980. When applicable, one was included for the evaluations made between 1991 & 1999. These were designed to capture unobserved effects, like nitrogen deposition.	
Latitude	Variable indicates "the approximate latitude of the plot".	
Longitude	Variable indicates "the approximate longitude of the plot".	

Supplementary Table 3. Stocking levels by forest group in southern states for all stands aged 1 to 100**.** States are AL, AR, FL, GA, LA, MS, NC, OK, SC, TN & TX using evaluation closest to 1974 (range: 1968 to 1980) and 2017 (range: 2017 to 2017).

Forest Group		White/Red/ Jack Pine	Spruce/ Fir	Slash/ Longleaf	Loblolly/ Shortleaf	Oak/ Pine	Oak/ Hickory	Oak/Gum/ Cypress	Elm/Ash/ Cottonwood	Maple/Beech/ Birch
Trees per Hectare	1974	356.3	586.4	262.7	351.3	259.6	226.2	273.3	215.9	295.7
	2017	456.0	758.3	336.8	413.9	243.8	212.4	242.6	142.0	215.1
	Δ (%)	28.0	29.3	28.2	17.8	-6.1	-6.1	-11.2	-34.2	-27.3

Supplementary Table 4. Stocking levels by forest group in northern states for all stands aged 1 to 100**.** States are CT, DE, IA, IL, IN, KS, KY, MA, MD, ME, MI, MN, MO, ND, NE, NH, NJ, NY, OH, PA, RI, SD, VA, VT, WI & WV using evaluation closest to 1985 (range: 1977 to 1993) and 2017 (range: 2017 to 2017).

Forest Group		White/Red/ Jack Pine	Spruce/ Fir	Loblolly/ Shortleaf	Oak/ Pine	Oak/ Hickory	Oak/Gum/ Cypress	Elm/Ash/ Cottonwood	Maple/Beech/ Birch	Aspen/ Birch
Trees per Hectare	1985	446.9	450.5	406.3	341.5	287.8	325.0	249.0	336.8	342.9
	2017	436.4	464.0	516.9	320.1	272.1	332.5	239.0	337.8	341.5
	Δ (%)	-2.3	3.0	27.2	-6.3	-5.4	2.3	-4.0	0.3	-0.4

Supplementary Table 5. Stocking levels by forest group in eastern United States for all stands aged 1 to 100. States are those east of the 100th meridian using evaluation closest to 1985 (range: 1980 to 1995) and 2017 (range: 2017 to 2017).

Forest Group		White Red Jack	Spruce Fir	Slash Longleaf	Loblolly Shortleaf	Oak Pine	Oak Hickory	Oak Gum Cypress	Elm Ash Cottonwood	Maple Beech Birch	Aspen Birch
Trees per Hectare	1985	448.1	451.1	321.9	373.0	276.1	267.3	311.1	244.3	337.0	346.7
	2017	437.0	464.3	336.5	422.1	266.2	248.0	247.2	202.9	336.7	341.5
	Δ (%)	-2.5	2.9	4.5	13.2	-3.6	-7.2	-20.5	-16.9	-0.1	-1.5

Supplementary Table 6. Stocking levels by forest group in 48 conterminous states for all stands aged 1 to 100**.** States are all of the 48 coterminous US states using evaluation closest to 1985 (range: 1980 to 1993) and 2017 (range: 2017 to 2017).

Forest Group		White Red Jack	Spruce Fir	Slash Longleaf	Loblolly Shortleaf	Oak Pine	Oak Hickory	Oak Gum Cypress	Elm Ash Cottonwood	Maple Beech Birch	Aspen Birch
Trees per Hectare	1985	448.1	451.1	321.9	373.0	276.1	267.3	311.1	243.0	337.0	373.7
	2017	437.0	464.2	336.5	422.1	266.2	248.0	247.2	203.1	336.7	345.9
	Δ (%)	-2.5	2.9	4.5	13.2	-3.6	-7.2	-20.5	-16.4	-0.1	-7.4

Supplementary Table 7. Change in predicted wood volume due to elevated CO_2 at ages 25/50/75 and due to other episodic phenomena from 1970 to 2015 using observations of naturally regenerated stands in the Eastern United States aged 1-100. Matches were created using observations of naturally regenerated plots in the Eastern U.S. with the control being observations from 1968-90 and the treatment being observations from 2000-18. States in the Eastern U.S. are: AL, AR, CT, DE, FL, GA, IL, IN, IA, KS, KY, ME, MD, MA, MI, MN, MS, MO, NH, NJ, NY, NC, OH, OK, PA, RI, SC, TN, TX, VT, VA, WV, and WI. Post-matching, full multivariate regression analysis was performed and then the effect of carbon fertilization was analyzed using a one-sided t-test to assess the difference in wood volume at each age given the age-specific CO_2 exposure for 1970 and 2015 (i.e. a 25-yr-old stand in 1970 received the sum of yearly exposure values from 1946 to 1970 and a 25-yr-old stand in 2015 received the sum of yearly exposure values from 1991 to 2015). Climate variables were held at their 1970 levels. A 99% confidence interval is shown for all but Spruce/Fir at 75 years of age where a 95% CI is shown. The impact of the other episodic phenomena was captured by the dummy variable comparing observations pre-1990 with those post-2000. Supplementary Data 24-25 show regression results. ***p<0.01, **p<0.05 and *p<0.10.

		Carbon Fertilization							Episodic Phenomena	Obs
		25 years old		50 years old		75 years old			Mean Impact	
Forest Type		Mean	CI	Mean	CI	Mean	CI		Post-2000 v. Pre-1990	#
White/Red/Jack Pine	Δ (%)	23.6	(9.7, 37.6)	18.0	(4.0, 31.9)	14.4	(0.4, 28.3)	Δ (%)	21.2	2,362
	Δ (m^3/ha)	12.9	(5.3, 20.6)	18.3	(4.1, 32.5)	26.3	(0.7, 51.9)	Sig.	***	
Spruce/Fir	Δ (%)	17.2	(5.1, 29.3)	13.1	(1.0, 25.2)	10.5	(1.9, 19.0)	Δ (%)	14.1	4,974
	Δ (m^3/ha)	6.0	(1.8, 10.2)	8.9	(0.7, 17.2)	9.8	(1.8, 17.9)	Sig.		
Slash/Longleaf Pine	Δ (%)	21.8	(10.3, 33.3)	16.6	(5.0, 28.1)	13.2	(1.7, 24.8)	Δ (%)	-9.8	3,582
	Δ (m^3/ha)	11.2	(5.3, 17.2)	17.3	(5.3, 29.3)	22.5	(2.9, 42.1)	Sig.	**	
Loblolly/Shortleaf Pine	Δ (%)	21.9	(17.1, 26.8)	16.7	(11.8, 21.5)	13.3	(8.5, 18.2)	Δ (%)	-2.3	15,188
	Δ (m^3/ha)	17.5	(13.6, 21.3)	31.1	(22.1, 40.1)	40.1	(25.5, 54.6)	Sig.		
Oak/Pine	Δ (%)	21.5	(15.9, 27.2)	16.3	(10.7, 22.0)	13.1	(7.4, 18.7)	Δ (%)	-3.2	9,670
	Δ (m^3/ha)	12.5	(9.2, 15.8)	20.0	(13.1, 26.9)	26.7	(15.2, 38.2)	Sig.		
Oak/Hickory	Δ (%)	19.0	(16.4, 21.5)	14.4	(11.9, 17.0)	11.5	(9.0, 14.1)	Δ (%)	-7.3	38,782
	Δ (m^3/ha)	11.1	(9.6, 12.6)	16.0	(13.2, 18.8)	19.6	(15.3, 23.9)	Sig.	***	
Oak/Gum/Cypress	Δ (%)	22.4	(16.6, 28.1)	17.0	(11.2, 22.8)	13.6	(7.8, 19.4)	Δ (%)	-2.9	11,446
	Δ (m^3/ha)	17.7	(13.2, 22.3)	25.3	(16.7, 33.8)	33.0	(19.0, 47.0)	Sig.		
Elm/Ash/Cottonwood	Δ (%)	18.0	(11.8, 24.3)	13.7	(7.5, 19.9)	11.0	(4.7, 17.2)	Δ (%)	-19.5	6,780
	Δ (m^3/ha)	10.5	(6.8, 14.1)	16.1	(8.8, 23.4)	18.0	(7.8, 28.3)	Sig.	***	
Maple/Beech/Birch	Δ (%)	16.5	(11.9, 21.2)	12.6	(7.9, 17.2)	10.0	(5.4, 14.7)	Δ (%)	-0.4	12,416
	Δ (m^3/ha)	10.1	(7.3, 13.0)	13.8	(8.6, 18.9)	15.8	(8.4, 23.2)	Sig.		
Aspen/Birch	Δ (%)	20.4	(16.0, 24.8)	15.5	(11.1, 19.9)	12.4	(8.0, 16.8)	Δ (%)	-12.7	16,404
	Δ (m^3/ha)	9.6	(7.5, 11.6)	14.7	(10.6, 18.8)	18.1	(11.7, 24.4)	Sig.	**	
All Forest Groups	Δ (%)	20.5	(16.1, 24.8)	15.6	(11.2, 19.9)	12.4	(8.1, 16.8)	Δ (%)	-9.7	121,604
	Δ (m^3/ha)	11.8	(9.3, 14.3)	18.6	(13.4, 23.8)	23.3	(15.2, 31.5)	Sig.	***	

Supplementary Table 8. Change in predicted volume (m^3ha^{-1}) between control and treatment groups at ages 25/50/75 using observations of naturally regenerated stands aged 1-100. Post-matching, nonlinear regression analysis was performed on observations of naturally regenerated plots and a comparison was made between the volume in the control (low CO_2) and treatment (high CO_2) periods at each age.

	25 years old	50 years old	75 years old
White/Red/Jack	27.6	92.7	121.7
Spruce/Fir	15.4	23.2	33.1
Slash/Longleaf	6.2	29.2	55.3
Loblolly/Shortleaf	24.0	37.0	35.5
Oak/Pine	9.2	17.3	48.9
Oak/Hickory	2.5	14.0	42.9
Oak/Gum/Cypress	7.6	21.3	9.1
Elm/Ash/Cottonwood	-3.8	17.4	25.2
Maple/Beech/Birch	-0.4	26.0	34.4
Aspen/Birch	-21.6	-6.9	-2.8

Supplementary Table 9. Change in predicted volume (%) for all forest groups due to elevated CO_2 at ages 25/50/75 from 1970 to 2015 using observations of naturally regenerated stands aged 1-100. Post-matching, nonlinear regression analysis was performed on observations of naturally regenerated plots and the effect of carbon fertilization was estimated by comparing the volume at each age given the age-specific CO_2 exposure for 1970 and 2015 (i.e. a 25-yr-old stand in 1970 received the sum of yearly exposure values from 1946 to 1970 and a 25-yr-old stand in 2015 received the sum of yearly exposure values from 1991 to 2015).

	25 years old	50 years old	75 years old
Δ (%)	27.3	25.9	11.3

Supplementary Table 10. Comparison of parameter on the natural log of lifetime CO_2 exposure variable using stands aged 1-100 and using stands aged 1-50

Observations used		Loblolly/Shortleaf	Longleaf/Slash	White/Red/Jack
Ages 1-100	Parameter	1.292	1.267	1.381
	95% CI	(1.250, 1.334)	(1.163, 1.371)	(1.258, 1.505)
Ages 1-50	Parameter	1.659	1.249	1.695
	95% CI	(1.585, 1.734)	(1.080, 1.419)	(1.488, 1.902)
