# Supplementary material for: The effect of carbon fertilization on naturally regenerated and planted US forests
Source: Nat Commun. 2022 Sep 19;13:5490. doi: 10.1038/s41467-022-33196-x (PMC9485135; doi:10.1038/s41467-022-33196-x)
Supplement: Supplementary file 2 — Reporting Summary [file 41467_2022_33196_MOESM2_ESM.pdf]

nature portfolio

Corresponding author(s): Davis, Eric

Last updated by author(s): Aug 24, 2022

Reporting Summary

Nature Portfolio wishes to improve the reproducibility of the work that we publish. This form provides structure for consistency and transparency in reporting. For further information on Nature Portfolio policies, see our Editorial Policies and the Editorial Policy Checklist.

Statistics

For all statistical analyses, confirm that the following items are present in the figure legend, table legend, main text, or Methods section.

n/a	Confirmed	
☐	☒	The exact sample size (*n*) for each experimental group/condition, given as a discrete number and unit of measurement
☐	☒	A statement on whether measurements were taken from distinct samples or whether the same sample was measured repeatedly
☐	☒	The statistical test(s) used AND whether they are one- or two-sided<br>*Only common tests should be described solely by name; describe more complex techniques in the Methods section.*
☐	☒	A description of all covariates tested
☐	☒	A description of any assumptions or corrections, such as tests of normality and adjustment for multiple comparisons
☐	☒	A full description of the statistical parameters including central tendency (e.g. means) or other basic estimates (e.g. regression coefficient) AND variation (e.g. standard deviation) or associated estimates of uncertainty (e.g. confidence intervals)
☐	☒	For null hypothesis testing, the test statistic (e.g. *F*, *t*, *r*) with confidence intervals, effect sizes, degrees of freedom and *P* value noted<br>*Give P values as exact values whenever suitable.*
☒	☐	For Bayesian analysis, information on the choice of priors and Markov chain Monte Carlo settings
☒	☐	For hierarchical and complex designs, identification of the appropriate level for tests and full reporting of outcomes
☒	☐	Estimates of effect sizes (e.g. Cohen's *d*, Pearson's *r*), indicating how they were calculated

Our web collection on statistics for biologists contains articles on many of the points above.

Software and code

Policy information about availability of computer code

Data collection	Data were derived from many sources. Forest growth data as well as information on the physical environment of the plots were obtained from the US Forest Service Forest Inventory and Analysis (USFS-FIA) Program. Precipitation and temperature data were obtained from the PRISM Climate Group. NOAA data were used for atmospheric CO2.
Data analysis	Code was written in R (version 4.1.2) to download and join data from USFS-FIA and the PRISM Climate Group and to create balanced matches using a supercomputer. Code was written in Stata 14.0 to join the NOAA data and to run the post-matching regressions.

For manuscripts utilizing custom algorithms or software that are central to the research but not yet described in published literature, software must be made available to editors and reviewers. We strongly encourage code deposition in a community repository (e.g. GitHub). See the Nature Portfolio guidelines for submitting code & software for further information.

Data

Policy information about availability of data

All manuscripts must include a data availability statement. This statement should provide the following information, where applicable:

- Accession codes, unique identifiers, or web links for publicly available datasets
- A description of any restrictions on data availability
- For clinical datasets or third party data, please ensure that the statement adheres to our policy

The data are all publicly available. All of the code will be made available on the website of one of the authors when published.

Field-specific reporting

Please select the one below that is the best fit for your research. If you are not sure, read the appropriate sections before making your selection.

☐ Life sciences ☐ Behavioural & social sciences ☒ Ecological, evolutionary & environmental sciences

For a reference copy of the document with all sections, see nature.com/documents/nr-reporting-summary-flat.pdf

Ecological, evolutionary & environmental sciences study design

All studies must disclose on these points even when the disclosure is negative.

Study description	This study estimates the effect of carbon fertilization and other time dependent factors on forest biomass from 1970 to 2015 while controlling for climate and other site characteristics using data from the USFS-FIA and the PRISM Climate Group.
Research sample	Forest plot data from the USFS-FIA database, which is freely available online and designed to be representative of a population that consists of the forest and rangelands of the United States, was utilized. All observations from evaluations conducted from the 1960s to 2018 were utilized, as these should have given a representative picture of the conditions across these years. These data were chosen because the observations cover a large number of years over which forested plots were exposed to different levels of CO2 and because a large number of plots were sampled allowing us to control for climate and other site specific characteristics that could influence the results. Only forest groups with more than 5,000 unmatched observations were considered to ensure there was sufficient statistical power to detect significant associations. With raw data chosen, we next attempted to approximate a random sample through matching before conducting estimations on naturally regenerated and planted stands for age classes ranging from 1 to 100.
Sampling strategy	Matching was performed to ensure results closer to those that would be obtained were a randomized controlled trial possible. Plots from the 2000 to 2018 period were matched without replacement with plots from the 1968 to 1990 period with balance created using a list of salient covariates and caliper widths less than or equal to 0.2 standard deviations of the propensity score in order to remove at least 98% of bias. All matches meeting these requirements were used in the analysis to generate as large a sample as possible. Observations were not selected from the 10-year period between 1990 and 2000 in order to allow for a minimum 10-year difference in observed CO2 concentration experienced by stands between the treatment and control samples.
Data collection	Data were obtained by Davis using code written in R (version 4.1.2) to download and join data from USFS-FIA and the PRISM Climate Group. Code written in Stata 14.0 was also used to join this data with the NOAA atmospheric data.
Timing and spatial scale	US Forest Service evaluations for individual states are conducted over a period of as many as five years and compiled into an evaluation group record for a designated year (e.g. Minnesota 2003 record was collected between 1999 and 2003). Specific sample dates are selected by the USFS and inventory personnel visit the site to collect data. Observations from before 2000 were obtained from periodic evaluations while observations after 2000 were obtained with annual evaluations. The spatial scale is the conterminous United States, although most of the data is obtained from the eastern part of the United States.
Data exclusions	Data for many forest types in the western US were excluded from the sample because there were not sufficient inventory evaluations in the pre-1990 period to allow us to control for elevated CO2.
Reproducibility	We conducted a number of robustness checks with the data, as noted in the supplemental appendix. The code is available for other researchers to estimate the results.
Randomization	The treatment group observations were matched with control group observations using observables such as climate, age, soil, slope, etc. Thus, each observation from the control group has an associated observation in the treatment group that is nearly identical except for the level of carbon dioxide it received over its lifetime.
Blinding	N/A--No human participants. Researchers were not blinded due to the need to know treatment status in order to facilitate matching to approximate a randomized controlled trial.

Did the study involve field work? ☐ Yes ☒ No

Reporting for specific materials, systems and methods

We require information from authors about some types of materials, experimental systems and methods used in many studies. Here, indicate whether each material, system or method listed is relevant to your study. If you are not sure if a list item applies to your research, read the appropriate section before selecting a response.

Materials & experimental systems

n/a	Involved in the study
☒	☐ Antibodies
☒	☐ Eukaryotic cell lines
☒	☐ Palaeontology and archaeology
☒	☐ Animals and other organisms
☒	☐ Human research participants
☒	☐ Clinical data
☒	☐ Dual use research of concern

Methods

n/a	Involved in the study
☒	☐ ChIP-seq
☒	☐ Flow cytometry
☒	☐ MRI-based neuroimaging
