# Supplementary material for: The effect of carbon fertilization on naturally regenerated and planted US forests
Source: Nat Commun. 2022 Sep 19;13:5490. doi: 10.1038/s41467-022-33196-x (PMC9485135; doi:10.1038/s41467-022-33196-x)
Supplement: Supplementary file 4 — Description of Additional Supplementary Files [file 41467_2022_33196_MOESM4_ESM.pdf]

File name: Supplementary Data 1-33, Supplementary Data 1
Description: Balance statistics summary by forest group using observations of naturally regenerated stands aged 1 to 100 with matches being between a pre-1990 control and a post-2000 treatment group. The control observations spanned 1968 to 1990 and the treatment observations were from 2000 to 2018.

File name: Supplementary Data 1-33, Supplementary Data 2
Description: Balance statistics summary by forest group using observations of naturally regenerated stands aged 1 to 100 with matches being between a pre-1990 control and a post-2000 treatment group. The control observations spanned 1968 to 1990 and the treatment observations were from 2000 to 2018.

File name: Supplementary Data 1-33, Supplementary Data 3
Description: Regression output using observations of naturally regenerated Loblolly/Shortleaf Pine stands aged 1 to 100. Results are of two-sided t tests of significance after matching (genetic matching and propensity score matching: nearest-neighbor with replacement) and after using a pooled regression on the raw data (unmatched sample). ***$p<0.01$, **$p<0.05$ and *$p<0.10$.

File name: Supplementary Data 1-33, Supplementary Data 4
Description: Regression output using observations of naturally regenerated Oak/Hickory stands aged 1 to 100. Results are of two-sided t tests of significance after matching (genetic matching and propensity score matching: nearest-neighbor with replacement) and after using a pooled regression on the raw data (unmatched sample). ***$p<0.01$, **$p<0.05$ and *$p<0.10$.

File name: Supplementary Data 1-33, Supplementary Data 5
Description: Post-matching raw regression output where the lifetime CO_2 variable captures the effect of carbon fertilization on all forests using observations of naturally regenerated stands aged 1 to 100. Results are of two-sided t tests of significance using all matched data from Supplementary Data 1 and Supplementary Data 2: control 1968-90 and treatment 2000-18. ***$p<0.01$, **$p<0.05$ & *$p<0.10$.

File name: Supplementary Data 1-33, Supplementary Data 6
Description: Post-matching raw regression output where the logged lifetime CO_2 variable captures the effect of carbon fertilization on all forests using observations of naturally regenerated stands aged 1 to 100. Results are of two-sided t tests of significance using all matched data from Supplementary Data 1 and Supplementary Data 2: control 1968-90 and treatment 2000-18. ***$p<0.01$, **$p<0.05$ & *$p<0.10$.

File name: Supplementary Data 1-33, Supplementary Data 7
Description: Regression output testing a logged lifetime CO_2 variable and various polynomial climate formulations for all forest groups using observations of naturally regenerated stands aged 1 to 100. Results are of two-sided t tests of significance using all matched data from Supplementary Data 1 and Supplementary Data 2: control 1968-90 and treatment 2000-18. ***$p<0.01$, **$p<0.05$ & *$p<0.10$.

File name: Supplementary Data 1-33, Supplementary Data 8

Description: Regression output testing a linear lifetime CO_2 variable and various polynomial climate formulations for all forest groups using observations of naturally regenerated stands aged 1 to 100. Results are of two-sided t tests of significance using all matched data from Supplementary Data 1 and Supplementary Data 2: control 1968-90 and treatment 2000-18. ***p<0.01, **p<0.05 & *p<0.10.

File name: Supplementary Data 1-33, Supplementary Data 9
Description: Regression output testing a logged lifetime CO_2 variable and additional climate formulations for all forest groups using observations of naturally regenerated stands aged 1 to 100. Results are of two-sided t tests of significance using all matched data from Supplementary Data 1 and Supplementary Data 2: control 1968-90 and treatment 2000-18. ***p<0.01, **p<0.05 & *p<0.10.

File name: Supplementary Data 1-33, Supplementary Data 10
Description: Regression output testing a linear lifetime CO_2 variable and additional climate formulations for all forest groups using observations of naturally regenerated stands aged 1 to 100. Results are of two-sided t tests of significance using all matched data from Supplementary Data 1 and Supplementary Data 2: control 1968-90 and treatment 2000-18. ***p<0.01, **p<0.05 & *p<0.10.

File name: Supplementary Data 1-33, Supplementary Data 11
Description: Key regression formulations used to create figures and tables

File name: Supplementary Data 1-33, Supplementary Data 12
Description: Post-matching raw regression output where the lifetime CO_2 variable captures the effect of carbon fertilization on Spruce/Fir using observations of naturally regenerated stands aged 1 to 100. Results are of two-sided t tests of significance using matched data of observations from naturally regenerated plots from Supplementary Data 1: control 1968-90 and treatment 2000-18. Model 1 results were used to create Table 1. Model 2 tested an alternate approach to capturing the impact of underlying, unobservable systematic differences like nitrogen deposition. Model 3 is similar to Model 1 with the impact of VPD and growing-season variables removed. Model 4 tested the impact on the CO_2 coefficient when site and climate variables were removed. ***p<0.01, **p<0.05 and *p<0.10.

File name: Supplementary Data 1-33, Supplementary Data 13
Description: Post-matching raw regression output where the lifetime CO_2 variable captures the effect of carbon fertilization on Oak/Gum/Cypress using observations of naturally regenerated stands aged 1 to 100. Results are of two-sided t tests of significance using matched data of observations from naturally regenerated plots from Supplementary Data 1: control 1968-90 and treatment 2000-18. Model 1 results were used to create Table 1. Model 2 tested an alternate approach to capturing the impact of underlying, unobservable systematic differences like nitrogen deposition. Model 3 is similar to Model 1 with the impact of VPD and growing-season variables removed. Model 4 tested the impact on the CO_2 coefficient when site and climate variables were removed. ***p<0.01, **p<0.05 and *p<0.10.

File name: Supplementary Data 1-33, Supplementary Data 14

Description: Post-matching raw regression output where the lifetime CO_2 variable captures the effect of carbon fertilization on Oak/Hickory using observations of naturally regenerated stands aged 1 to 100. Results are of two-sided t tests of significance using matched data of observations from naturally regenerated plots from Supplementary Data 1: control 1968-90 and treatment 2000-18. Model 1 results were used to create Table 1. Model 2 tested an alternate approach to capturing the impact of underlying, unobservable systematic differences like nitrogen deposition. Model 3 is similar to Model 1 with the impact of VPD and growing-season variables removed. Model 4 tested the impact on the CO_2 coefficient when site and climate variables were removed. ***p<0.01, **p<0.05 and *p<0.10.

File name: Supplementary Data 1-33, Supplementary Data 15
Description: Post-matching raw regression output where the lifetime CO_2 variable captures the effect of carbon fertilization on Oak/Pine using observations of naturally regenerated stands aged 1 to 100. Results are of two-sided t tests of significance using matched data of observations from naturally regenerated plots from Supplementary Data 1: control 1968-90 and treatment 2000-18. Model 1 results were used to create Table 1. Model 2 tested an alternate approach to capturing the impact of underlying, unobservable systematic differences like nitrogen deposition. Model 3 is similar to Model 1 with the impact of VPD and growing-season variables removed. Model 4 tested the impact on the CO_2 coefficient when site and climate variables were removed. ***p<0.01, **p<0.05 and *p<0.10.

File name: Supplementary Data 1-33, Supplementary Data 16
Description: Post-matching raw regression output where the lifetime CO_2 variable captures the effect of carbon fertilization on Maple/Beech/Birch using observations of naturally regenerated stands aged 1 to 100. Results are of two-sided t tests of significance using matched data of observations from naturally regenerated plots from Supplementary Data 1: control 1968-90 and treatment 2000-18. Model 1 results were used to create Table 1. Model 2 tested an alternate approach to capturing the impact of underlying, unobservable systematic differences like nitrogen deposition. Model 3 is similar to Model 1 with the impact of VPD and growing-season variables removed. Model 4 tested the impact on the CO_2 coefficient when site and climate variables were removed. ***p<0.01, **p<0.05 and *p<0.10.

File name: Supplementary Data 1-33, Supplementary Data 17
Description: Post-matching raw regression output where the lifetime CO_2 variable captures the effect of carbon fertilization on Elm/Ash/Cottonwood using observations of naturally regenerated stands aged 1 to 100. Results are of two-sided t tests of significance using matched data of observations from naturally regenerated plots from Supplementary Data 1: control 1968-90 and treatment 2000-18. Model 1 results were used to create Table 1. Model 2 tested an alternate approach to capturing the impact of underlying, unobservable systematic differences like nitrogen deposition. Model 3 is similar to Model 1 with the impact of VPD and growing-season variables removed. Model 4 tested the impact on the CO_2 coefficient when site and climate variables were removed. ***p<0.01, **p<0.05 and *p<0.10.

File name: Supplementary Data 1-33, Supplementary Data 18

Description: Post-matching raw regression output where the lifetime CO_2 variable captures the effect of carbon fertilization on Aspen/Birch using observations of naturally regenerated stands aged 1 to 100. Results are of two-sided t tests of significance using matched data of observations from naturally regenerated plots from Supplementary Data 1: control 1968-90 and treatment 2000-18. Model 1 results were used to create Table 1. Model 2 tested an alternate approach to capturing the impact of underlying, unobservable systematic differences like nitrogen deposition. Model 3 is similar to Model 1 with the impact of VPD and growing-season variables removed. Model 4 tested the impact on the CO_2 coefficient when site and climate variables were removed. ***p<0.01, **p<0.05 and *p<0.10.

File name: Supplementary Data 1-33, Supplementary Data 19
Description: Post-matching raw regression output where the lifetime CO_2 variable captures the effect of carbon fertilization on White/Red/Jack Pine using observations of naturally regenerated stands aged 1 to 100. Results are of two-sided t tests of significance using matched data of observations from naturally regenerated plots from Supplementary Data 1: control 1968-90 and treatment 2000-18. Model 1 results were used to create Table 1. Model 2 tested an alternate approach to capturing the impact of underlying, unobservable systematic differences like nitrogen deposition. Model 3 is similar to Model 1 with the impact of VPD and growing-season variables removed. Model 4 tested the impact on the CO_2 coefficient when site and climate variables were removed. ***p<0.01, **p<0.05 and *p<0.10.

File name: Supplementary Data 1-33, Supplementary Data 20
Description: Post-matching raw regression output where the lifetime CO_2 variable captures the effect of carbon fertilization on Loblolly/Shortleaf Pine using observations of naturally regenerated stands aged 1 to 100. Results are of two-sided t tests of significance using matched data of observations from naturally regenerated plots from Supplementary Data 1: control 1968-90 and treatment 2000-18. Model 1 results were used to create Table 1. Model 2 tested an alternate approach to capturing the impact of underlying, unobservable systematic differences like nitrogen deposition. Model 3 is similar to Model 1 with the impact of VPD and growing-season variables removed. Model 4 tested the impact on the CO_2 coefficient when site and climate variables were removed. ***p<0.01, **p<0.05 and *p<0.10.

File name: Supplementary Data 1-33, Supplementary Data 21
Description: Post-matching raw regression output where the lifetime CO_2 variable captures the effect of carbon fertilization on Slash/Longleaf Pine using observations of naturally regenerated stands aged 1 to 100. Results are of two-sided t tests of significance using matched data of observations from naturally regenerated plots from Supplementary Data 1: control 1968-90 and treatment 2000-18. Model 1 results were used to create Table 1. Model 2 tested an alternate approach to capturing the impact of underlying, unobservable systematic differences like nitrogen deposition. Model 3 is similar to Model 1 with the impact of VPD and growing-season variables removed. Model 4 tested the impact on the CO_2 coefficient when site and climate variables were removed. ***p<0.01, **p<0.05 and *p<0.10.

File name: Supplementary Data 1-33, Supplementary Data 22
Description: Post-matching raw regression output where the lifetime CO_2 variable captures the effect of carbon fertilization on all forests using observations of naturally regenerated stands aged 1 to 100. Results are of two-sided t tests of significance using matched data of observations from naturally regenerated plots from Supplementary Data 1: control 1968-90 and treatment 2000-18. Model 1 results were used to create Table 1. Model 2 tested an alternate approach to capturing the impact of underlying, unobservable systematic differences like nitrogen deposition. Model 3 is similar to Model 1 with the impact of VPD and growing-season variables removed. Model 4 tested the impact on the CO_2 coefficient when site and climate variables were removed. ***$p<0.01$, **$p<0.05$ and *$p<0.10$.

File name: Supplementary Data 1-33, Supplementary Data 23
Description: Balance statistics summary by forest group using observations of naturally regenerated stands from the Eastern U.S. aged 1 to 100 with matches being between a pre-1990 control and a post-2000 treatment group. The control observations spanned 1968 to 1990 and the treatment observations were from 2000 to 2018. States in the Eastern U.S. are: AL, AR, CT, DE, FL, GA, IL, IN, IA, KS, KY, ME, MD, MA, MI, MN, MS, MO, NH, NJ, NY, NC, OH, OK, PA, RI, SC, TN, TX, VT, VA, WV, and WI.

File name: Supplementary Data 1-33, Supplementary Data 24
Description: Post-matching raw regression output by forest group where the lifetime CO_2 variable captures the effect of carbon fertilization using observations of naturally regenerated stands aged 1 to 100 from the Eastern U.S. Results are of two-sided t tests of significance using matched data of observations from naturally regenerated plots in the Eastern U.S. from Supplementary Data 23: control 1968-90 and treatment 2000-18. ***$p<0.01$, **$p<0.05$ and *$p<0.10$.

File name: Supplementary Data 1-33, Supplementary Data 25
Description: Post-matching raw regression output on all forest groups where the lifetime CO_2 variable captures the effect of carbon fertilization using observations of naturally regenerated stands aged 1 to 100 from the Eastern U.S. Results are of two-sided t tests of significance using matched data of observations from naturally regenerated plots in the Eastern U.S. from Supplementary Data 2 and Supplementary Data 23: control 1968-90 and treatment 2000-18. ***$p<0.01$, **$p<0.05$ and *$p<0.10$.

File name: Supplementary Data 1-33, Supplementary Data 26
Description: Post-matching nonlinear raw regression output by forest group and by treatment status using observations of naturally regenerated stands aged 1 to 100. Results are of two-sided t tests of significance using post-matching observations of naturally regenerated plots. The control column used observations from the pre-1990 (low CO_2) period and the treatment column used observations from the post-2000 (high CO_2) period. ***$p<0.01$, **$p<0.05$ and *$p<0.10$.

File name: Supplementary Data 1-33, Supplementary Data 27
Description: Post-matching nonlinear raw regression output for all forest groups using observations of naturally regenerated stands aged 1 to 100. Results are of two-sided t tests of significance using post-matching observations of naturally regenerated plots. ***$p<0.01$, **$p<0.05$ and *$p<0.10$.

File name: Supplementary Data 1-33, Supplementary Data 28
Description: Balance statistics summary for White/Red/Jack pine by regrowth method using observations of stands aged 1 to 50 with matches being between a pre-1990 control and a post-2000 treatment group. The data were truncated to observations aged 1 to 50 years. Then matching occurred with control observations spanning 1968 to 1990 and treatment observations spanning from 2000 to 2018.

File name: Supplementary Data 1-33, Supplementary Data 29
Description: Balance statistics summary for Slash/Longleaf pine by regrowth method using observations of stands aged 1 to 50 with matches being between a pre-1990 control and a post-2000 treatment group. The data were truncated to observations aged 1 to 50 years. Then matching occurred with control observations spanning 1968 to 1990 and treatment observations spanning from 2000 to 2018.

File name: Supplementary Data 1-33, Supplementary Data 30
Description: Balance statistics summary for Loblolly/Shortleaf pine by regrowth method using observations of stands aged 1 to 50 with matches being between a pre-1990 control and a post-2000 treatment group. The data were truncated to observations aged 1 to 50 years. Then matching occurred with control observations spanning 1968 to 1990 and treatment observations spanning from 2000 to 2018.

File name: Supplementary Data 1-33, Supplementary Data 31
Description: Post-matching raw regression output for White/Red/Jack pine by regrowth method using observations of naturally regenerated stands aged 1 to 50 and where the lifetime CO_2 variable captures the effect of carbon fertilization. Results are of two-sided t tests of significance using matched data of observations from naturally regenerated and planted plots in Supplementary Data 28: control (1968-90) and treatment (2000-18). ***$p<0.01$, **$p<0.05$ and *$p<0.10$.

File name: Supplementary Data 1-33, Supplementary Data 32
Description: Post-matching raw regression output for Slash/Longleaf pine by regrowth method using observations of naturally regenerated stands aged 1 to 50 and where the lifetime CO_2 variable captures the effect of carbon fertilization. Results are of two-sided t tests of significance using matched data of observations from naturally regenerated and planted plots in Supplementary Data 29: control (1968-90) and treatment (2000-18). ***$p<0.01$, **$p<0.05$ and *$p<0.10$.

File name: Supplementary Data 1-33, Supplementary Data 33
Description: Post-matching raw regression output for Loblolly/Shortleaf pine by regrowth method using observations of naturally regenerated stands aged 1 to 50 and where the lifetime CO2 variable captures the effect of carbon fertilization. Results are of two-sided t tests of significance using matched data of observations from naturally regenerated and planted plots in Supplementary Data 30: control (1968-90) and treatment (2000-18). ***$p<0.01$, **$p<0.05$ and *$p<0.10$.
